# Suicidal Crisis among Children and Young People: Associations with Adverse Childhood Experiences and Socio-Demographic Factors

**DOI:** 10.3390/ijerph20021251

**Published:** 2023-01-10

**Authors:** Emma Ashworth, Ian Jarman, Philippa McCabe, Molly McCarthy, Serena Provazza, Vivienne Crosbie, Zara Quigg, Pooja Saini

**Affiliations:** 1 Faculty of Health, Liverpool John Moores University, Liverpool L3 3AF, UK; 2 Faculty of Engineering and Technology, Liverpool John Moores University, Liverpool L3 3AF, UK; 3 Alder Hey Children’s NHS Foundation Trust, Liverpool L14 5AB, UK

**Keywords:** children and young people, suicidal crisis, adverse childhood experiences, emergency departments

## Abstract

Suicide is a major public health issue and a leading cause of death among children and young people (CYP) worldwide. There is strong evidence linking adverse childhood experiences (ACEs) to an increased risk of suicidal behaviours in adults, but there is limited understanding regarding ACEs and suicidal crises in CYP. This study aims to examine the ACEs associated with CYP presenting at Emergency Departments for suicidal crises, and specifically the factors associated with repeat attendances. This is a case series study of CYP (aged 8–16) experiencing suicidal crisis who presented in a paediatric Emergency Department in England between March 2019 and March 2021 (n = 240). The dataset was subjected to conditional independence graphical analysis. Results revealed a significant association between suicidal crisis and several ACEs. Specifically, evidence of clusters of ACE variables suggests two distinct groups of CYP associated with experiencing a suicidal crisis: those experiencing “household risk” and those experiencing “parental risk”. Female sex, history of self-harm, mental health difficulties, and previous input from mental health services were also associated with repeat hospital attendances. Findings have implications for early identification of and intervention with children who may be at a heightened risk for ACEs and associated suicidal crises.

## 1. Introduction

Suicide is a major public health issue and is a leading cause of death among children and young people (CYP) worldwide [1]. In the last decade, there has been a 7.9% increase annually in suicide rates among adolescents in England and Wales [2]. Rates have been seen to increase further recently, with data from the Office of National Statistics [3] reporting a 22% one-year increase in suicide rates for those under 25 years old, the largest rise amongst all age groups [4]. Suicidal crisis, defined as a spectrum ranging from thoughts about death with no intent or plan to die by suicide to specific suicidal ideation with an intent or plan, has been identified as a key risk factor for self-harm and future suicide attempts [5,6]. Indeed, research suggests that suicidal ideation often precedes a suicide attempt; for example, a longitudinal study in the US found that more than one-third of adolescents in suicidal crisis went on to attempt suicide [7]. Furthermore, it has been shown that the more pervasive the suicidal crisis, the more likely the individual is to attempt suicide [8]. In the UK, 42% of young adults reported having suicidal thoughts on at least one occasion in the previous twelve months [9]. Additionally, according to community samples, 5–42% of young people engage in non-suicidal self-injury [9,10] and suicidal thoughts are reported in 15–25% of CYP [11,12]. Given the high prevalence of suicidal crises among CYP, it is imperative that healthcare services, policies, and guidelines ensure suicide prevention among CYP is a key priority.

While suicidal crisis is a clear risk factor for later suicide attempts, little is known about the predictors of suicidal crisis among CYP. The Integrated Motivational-Volition Model of Suicidal Behaviour (IMV [13]) recognises the complex interplay of biological, psychological, environmental, and cultural factors in suicidal thoughts and behaviour. Several research studies have examined a range of predictors for suicidal thoughts; for example, Rohde et al. [14] investigated risk factors from six categories associated with suicide-related thoughts and/or behaviours for adolescents, which included: demographic factors, suicide/depression, problem behaviour factors, personality factors, parent/family factors, and peer factors. Similarly, Beautrais [15] noted that risk factors related to youth suicidal behaviour could be categorised into several domains including social and educational disadvantage, childhood and family adversity, psychopathology, individual and personal vulnerabilities, exposure to stressful life events and social, and cultural and contextual factors. More recent research [16,17,18] has also noted specific risk factors including feelings of loneliness, worthlessness, hopelessness and burdensomeness, impulsivity, psychosis symptoms, and behavioural problems. However, the majority of research tends to focus on an adult crisis or actual suicidal behaviours, failing to offer a nuanced understanding of suicidal crises within CYP.

### 1.1. Adverse Childhood Experiences and Suicidality

Adverse childhood experiences (ACEs) refer to traumatic events in the first 18 years of life. These include multiple types of child abuse and neglect, as well as other types of serious household dysfunction, such as alcohol and substance abuse, divorce/separation of parents, witnessing domestic violence, and family financial difficulties [19]. Numerous studies have shown that certain ACEs, for example child abuse and neglect, raise the risk of suicidal behaviours in adulthood [20]. Furthermore, according to a recent systematic review, there is strong evidence linking childhood maltreatment to an increased risk of suicidal behaviours in adults [21]. However, while suicide has long been recognised as a multifactorial issue, there is limited understanding regarding the complexities of ACEs and suicidal crises specifically among CYP [22].

Previous research into ACEs tends to explore the outcome measure of suicidal behaviours in adulthood. This limits research findings as their assessment of ACEs is taken retrospectively during adulthood, which has low concordance with the assessment of ACEs in childhood [23]. Few studies have examined the impact of ACEs specifically on suicidal thoughts and behaviours within CYP. However, one study by Miche et al. [24] involved a 10-year longitudinal community sample that examined suicidal behaviours and ACEs in adolescents and young adults aged 14–24 years. The risk of suicide attempts for this sample was demonstrated to increase in response to various traumatic events, with rape/sexual abuse having the highest hazard ratio [24]. Similarly, a school-based health survey carried out across four regions in China revealed that CYP aged between 10 and 20 years who encountered a higher number of ACEs were more likely to report suicidal behaviours than those who experiences fewer ACEs [25]. As such, consistent findings suggest a relationship between ACEs, suicidal behaviours, and suicide attempts (e.g., [26,27]). However, there is a paucity of research focused specifically on the relationship between ACEs and CYP’s suicidal thoughts, with even less research being conducted within a UK setting.

In addition, a common limitation of previous ACEs research has been the narrow definition and outcome measures used to explore adverse experiences. For instance, the initial ACEs study by Felitti et al. [28] explored 10 adverse experiences; five that involved direct harm to the child and five that affected the environment in which they grew up. This limited categorisation fails to account for additional adverse experiences and stressors CYP commonly encounter. For example, evidence exists for risk factors related to a sense of belonging [29,30], burdensomeness [31,32], and bullying [33,34], and subsequent suicidal behaviour in CYP. As such, it is vital that future research explores a wider range of socio-demographic variables and moves beyond the traditional ACEs, to better explore individual traits and situational variables associated with CYP suicidal crisis and suicide-related behaviours.

### 1.2. ACEs and Use of Healthcare Services

ACE-associated adverse health outcomes are common reasons for presentations to acute healthcare services [35]; in particular, they are associated with increased demand for Emergency Department (ED) services [36,37]. Compared to the general population, individuals experiencing ACEs have been shown to visit the ED more often [38]. For example, one study conducted in Canada reported a 29% increase in visits to EDs for those individuals with more than one ACE, compared to a general population sample without any reported ACEs [39]. Another study among children and adolescents indicated that those who reported childhood abuse had a significantly higher number of ED visits than their counterparts (2.1 vs. 1.5; [40]). It has also been reported that CYP with ACEs have higher healthcare utilisation costs, with a significant factor influencing these overall higher costs being increased ED visits [41]. A better understanding of ACEs, suicidal crises, and subsequent ED visits for CYP could have a significant positive impact on patients, as well as reducing the pressure and demand on health care services.

However, while ACEs research has commonly been used to inform public health promotion and prevention programmes, both clinicians and researchers have questioned the ability of ACEs research to predict healthcare events at the individual level, for example ED visits [38,42]. In terms of suicidality specifically, this challenge stems partly from the complexity of suicide (as it is rarely caused by a single factor, e.g., [13]), a lack of understanding regarding CYP suicidal crisis, and the limited categorisations utilised in ACEs research. To address the public health challenge of steadily increasing suicide rates among CYP, it is thus essential to better understand ACEs’ association with CYP suicidal ideation, in addition to other socio-demographic characteristics. Therefore, this exploratory study aims to build a better picture of CYP who experience suicidal crises, by examining the ACEs and socio-demographic characteristics associated with ED presentations at a local children’s hospital over a two-year period. Furthermore, given the association between the length of the suicidal crisis and future suicide attempts [8], the study aims to examine the factors associated with *repeated* attendance at ED for suicidal crisis; in other words, the ACEs and other socio-demographic characteristics associated with *pervasive and enduring* crises. Thus, the research questions for the current study are as follows:

1. Which ACEs are associated with children experiencing a suicidal crisis?
2. What are the factors associated with repeat ED attendance for children (i.e., multiple episodes of suicidal crisis)?

## 2. Materials and Methods

### 2.1. Design and Setting

This retrospective case series study included CYP experiencing a suicidal crisis who had attended an ED at a local paediatric hospital in North-West England between March 2019 and March 2021 (n = 240). Access to the anonymised data was approved by the hospital’s research department. The data collection methods utilized here have been published previously [4].

### 2.2. Participants and Data Extraction

Clinical records at the hospital were reviewed between March 2019 and March 2021. Inclusion criteria included any patients aged 16 or younger (the hospital advises anyone aged over 16 to attend an adult ED) who presented to ED in a suicide crisis (with and without self-harm) during the study period. Anonymised data on CYP who visited the hospital in suicidal crisis were provided to the researcher by the hospital data team. An electronic inspection of the clinical notes was performed through the Meditech system (Medical Information Technology Inc., Westwood, MA, USA). All patient notes under potentially relevant codes (e.g., low mood, suicide thoughts, social problems, overdose) were audited, and those indicating suicidal ideation were extracted, collated, and anonymised. Each patient’s clinical record was inspected and included in the study only if suicide ideation was clearly reported in the clinical notes.

Variables examined were mostly binary variables extracted from clinicians’ notes and included ACEs (physical abuse, emotional abuse, sexual abuse, neglect, exposure to domestic violence, parental engagement in criminality, parents’ drug misuse, parental mental ill health, separated parents), as well as demographic variables (e.g., sex, ethnicity, special educational needs [SEN], presence of suspected autism traits, mental health conditions, suicide ideation with or without self-harm, history of self-harm, clinician determined risk to life [in terms of Pierce Suicide Intent Scale score], and frequency of previous ED attendance for suicidal crisis [converted to binary 0/1 variable for this analysis]). It was also investigated whether the children were previously known to Child and Adolescent Mental Health Services (CAMHS) or were under CAMHS at the time of the ED presentation. These data were either collected from the family using a standard proforma completed by the clinician when triaging the patient, or they were already available on the hospital system if the patient had been previously open to or was currently known to community paediatrics or CAMHS.

### 2.3. Data Analysis

The dataset was subjected to conditional independence graphical analysis [43,44,45] where the algorithm systematically tests the statistical association between pairs of variables given knowledge about another variable, more technically referred to as conditional independence. Since mutual covariance is common in multivariate analyses—and especially common in the social sciences—the approach ensures that each specific test of association between variables is repeated and conditioned on all other variables considered in the analysis. The resulting multivariate association structure is represented in a conditional independence map (CI-Map). Any pair of variables that do not carry significant mutual information about each other are disconnected from the map. The remaining associations are then directed according to the relative strength of conditional probabilities and subjected also to additional constraints, so that the final map is consistent and acyclic, thus removing any closed loops [43,46]. “Mutual Information” (MI) provides a measure of strength of association between a pair of variables, compared to all other significant links in the CI-Map. Thus, using MI can provide a useful insight into the strength of associations, beyond that of simply stating a significant *p* value (i.e., *p* < 0.05). However, it must be stressed that the statistical links are associations based on the data sample and no causal link can be inferred based on the analysis.

By way of an analogy, the resulting CI-Map could be thought of as a “statistical mind map”, representing a visualisation of the full association structure of all the variables together in a single analysis. The CI-Map in this study takes in a variety of information about the ED visit, including ACEs and demographic information about the patient that are used to construct the “statistical mind map”. The CI-Map can be a vital tool to gain insights into the pathways and relationships between the variables which is completely data-driven and provides evidence-based understanding that can support decision-making.

## 3. Results

Between March 2019 and 2021, 240 CYP attended the hospital’s ED for suicidal crisis (see Table 1). Rates of self-harm were calculated using the first episode in the study period; hospital attendance prior to the study period and re-attendances during the two-year study period were also included. Comprehensive demographic details have previously been published [4]; however, to summarise, the majority of attendees were female (67%) and White British (93%), and the mean age was 13.5 (SD = 1.42; range = 8–16). Approximately one-quarter of CYP had a diagnosed SEN (24%; n = 58) and the majority had a diagnosed mental health condition (60%; n = 142), typically anxiety (44%) or low mood (32%). A total of 69% had a history of self-harm and 35% (n = 83) presented with suicidal ideation in addition to deliberate self-harm in the current data capture. For the majority of CYP, this was their first visit to ED for a suicidal crisis (76%; n = 183), although approximately one-quarter had attended previously (24%). Table 1 provides a summary of demographic characteristics by previous ED attendance for a suicidal crisis, and first attendance in the considered period (i.e., 2019–2021).

**Figure 1:**
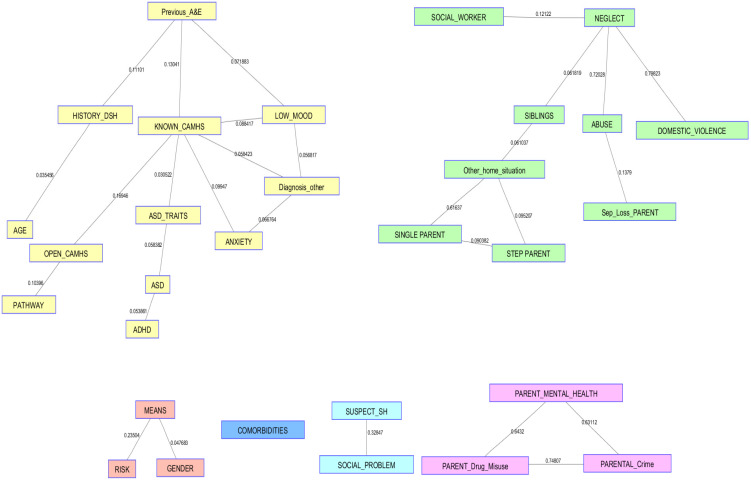
CI-Map.

The results of the analyses are summarised in the CI-Map (Figure 1), which shows variables that are significantly associated (*p* < 0.05), having tested for all possible associations. Each connecting line in Figure 1 therefore represents the associated pairwise MI between the respective variables. Higher MI corresponds to a stronger pairwise association; thus, for this map the strongest pairwise association is 0.80 and the weakest 0.05, but all are significant at the 95% confidence level.

Pairwise associations are calculated for every variable in the analysis. On inspection, it is clear that not all the data are linked, which is to say, there are groups of variables linked together that do not link to another set of variables. These distinct sets of linked variables reflect aspects specific to the person or family situation. For instance, the self-contained set of variables in the top left can be described as “person-centred”; this set is related to individual characteristics (low mood, anxiety, ASC) and these conditions have generally led to an interaction with CAMHS. If we move our attention to the next set of variables (top right), they are themed around the family and outcomes that can arise from this setting, for instance, neglect or domestic violence. Moving on to the bottom right, this shows a triangulation of parent-centred information around mental health, drug misuse, and crime. The final associations to highlight are the bottom left, which involve biological sex and the means of self-harm that have been used. All these themes need to be explored and understood at some level, which may entail further work.

Taking note of these different structures and inspecting the map in more detail reveals that the variable “previous ED attendance for suicidal crisis” was directly and significantly associated with a history of deliberate self-harm (*p* < 0.001, MI = 0.11), being previously known to CAMHS (*p* < 0.01, MI = 0.13), and having a diagnosed mental health condition in the low mood/depression category (*p* < 0.01, MI = 0.07). Given all the possible associations of “previous ED attendance” with every other variable in the data, it was only these three variables that conveyed statistically significant conditional MI. All the variables included in the analysis were represented on the map.

Further examination of the map reveals variables indirectly associated with previous ED attendance for suicidal crisis via being known to CAMHS. There are several variables directly associated with CAMHS that relate to either a neurodevelopmental or mental health condition, including suspected autistic traits (*p* < 0.01, MI = 0.03), and a diagnosis of anxiety *(p* < 0.01, MI = 0.10), low mood (*p* < 0.01, MI = 0.09), or other mental health condition (*p* < 0.01, MI = 0.06). This suggests that the young people who repeatedly present at the ED for a suicidal crisis are those who have already been under CAMHS services, for one of two key reasons: either because they had previously been referred to receive a diagnosis of an SEN, or because they had already been experiencing internalising mental health conditions.

Furthermore, the map shows several clusters of associated ACE variables. Firstly, and as already noted, there are several variables relating to family structure and home circumstances that that are significantly connected. In particular, neglect is statistically significantly associated with being under a social worker (*p* < 0.01, MI = 0.12) and experiencing domestic violence (*p* < 0.01, MI = 0.80) and abuse (*p* < 0.01, MI = 0.72) in the home. Neglect is also indirectly associated with familial structure, including living with single or stepparents, and the number of siblings in the home. This suggests that there is a significant cluster of CYP in the dataset who are living in home circumstances characterised by abuse, neglect, or violence.

Secondly, three ACE variables relating to the parents are clustered together; specifically, mental health difficulties, drug misuse, and engagement in crime all share significant MI. Thus, this shows that there is also a significant cluster of CYP in the dataset whose parents are experiencing difficulties with mental ill health, drug misuse, and/or criminality. Furthermore, the relationships between the variables suggest that parents who experience one of these difficulties may also be experiencing the other difficulties.

Finally, a significant cluster of variables is also present in the map specifically relating to CYP who present in the ED with suicide ideation in addition to deliberate self-harm. Specifically, means of self-harm is significantly associated with sex (*p* < 0.01, MI = 0.05) and risk to life (*p* < 0.01, MI = 0.24). Further analysis of the data indicates that females are significantly more likely to present with deliberate self-harm as well as suicidal ideation than males (X(1) = 6.23, *p* = 0.013, ϕ = 0.16) and, in particular, are significantly more likely to have overdosed compared to males (X(1) = 10.54, *p* = 0.001, ϕ = 0.21). There are no statistically significant sex differences for suffocation or cutting (although numbers in these categories are small). The level of risk to life assigned by the clinician is also associated with means of self-harm (X(8) = 25.0, *p* = 0.002, ϕ = 0.44), whereby CYP presenting with suicide ideation only are significantly more likely to be given a low-risk code (X(2) = 11.73, *p* = 0.003, ϕ = 0.30), while those who have overdosed are significantly more likely to be assigned a high-risk code (X(2) = 13.18, *p* = 0.001, ϕ = 0.32).

## 4. Discussions

This exploratory case series study aimed to build a picture of patients who presented at a paediatric ED for suicidal crisis, with the purpose of elucidating the factors that are present among CYP in suicidal crisis. More specifically, we hypothesised that ACEs, along with a range of other socio-demographic variables, would be associated with frequent ED attendance for suicidal crisis in young people. The relationship between ACEs and suicidality in adults is well-established and widely investigated; however, to the best of our knowledge, no extensive research has been carried out to investigate the relationship between ACEs and suicidal ideation in CYP.

The CI-Map revealed a significant association between suicidal crises and several ACEs. Specifically, evidence of clusters of ACE variables found in our study suggests two distinct groups of CYP associated with experiencing a suicidal crisis: those experiencing “household risk” and those experiencing “parental risk”. CYP appeared to either be exposed to ACEs relating to abuse, neglect, or violence (i.e., household factors; [26,47,48]), or to ACEs variables relating to their parents, such as parents’ mental illness, criminality, and drug misuse. The findings also suggest that a group of CYP in our sample were living in circumstances in which their household or parents may have presented with more than one difficulty at the same time. The clusters identified here are in keeping with the existing ACEs literature, which suggests that ACEs fall into one of two domains: factors that involve direct harm to the child, and factors that affect the environment in which they grow up [28]. However, it should be noted that ACEs in these two domains are often likely to co-occur; indeed, the findings in the present study do not preclude the family situation influencing the home situation, and vice versa, but instead suggest that for CYP presenting in the ED for suicidal crisis, these appear to be two separate sets of associations.

This study highlights the importance of taking ACEs into consideration when assessing suicidal crises in childhood, and adds further evidence of the potentially detrimental role of exposure to traumatic experiences in CYP. This finding also supports recent machine learning work which has sought to examine a large number of predictors for suicidality in CYP. For instance, one study found that while a significant pathway to suicidality in CYP is depression and a desire to die, another pathway exists whereby suicidality is integrally tied to frustration, reactive aggression, and poor impulse control, particularly in response to stressful life events [17], such as ACEs. The findings also emphasise the importance of early identification and intervention, through the implementation of effective programmes to reduce the number of ACEs to which children are exposed, and the provision of support for CYP who may be exposed to multiple household or parental ACEs. In turn, this will help to prevent the number of young people from reaching the point of crisis.

Furthermore, we found additional socio-demographic variables that were associated with suicidal crises in our sample of CYP. Specifically, the majority of CYP who attended the ED were females (48%), had previously self-harmed (44.6%), were diagnosed with mental health difficulties (27.5%), and had previously received input from mental health services (29%). Particularly noteworthy is that one-quarter of CYP who visited the ED in a suicidal crisis had a history of hospital attendance for the same reason (24%). A history of previous admissions for a suicidal crisis was higher in those who had a history of self-harm (23%) and those who were previously under CAMHS (27%). The relationship between the history of ED attendance for a suicidal crisis, history of self-harm, and being known to CAMHS was confirmed by the CI-Map, which revealed that the variable “previous ED attendance” was directly and significantly associated with a history of self-harm and being previously under CAMHS.

Typically, in the UK, young people may have been known to CAMHS for either a diagnosis of mental health difficulties or for an SEN diagnosis (e.g., autism and ADHD), and both of these were significantly associated with previously being known to CAMHS in the present study. Mental health difficulties relating to low mood were significantly and directly associated with previous ED attendance for a suicidal crisis, which is perhaps unsurprising, and confirms that low mood/depression plays a potential role in suicidality [49,50,51]. Remarkably however, no relationship between SEN and mental health difficulties was found in this study, despite evidence from previous literature of comorbidity between SEN (especially autism) and mental health difficulties (see, e.g., [4,52,53]). One potential reason for this may be explained in terms of diagnostic overshadowing [54]. This refers to practitioners’ tendencies to attribute individuals’ symptoms or behaviours to their existing diagnosis (e.g., autism), resulting in underdiagnosis of comorbid conditions (e.g., mental health conditions). In the present study, children who had already received a diagnosis of an SEN may have presented with comorbid mental health conditions which then went underdiagnosed, with any symptoms being attributed instead to their SEN. Consequently, these children may not have received the correct support, resulting in their difficulties increasing to the point of crisis, thus leading to their presentation to the ED. Therefore, it is vital that clinicians acknowledge that CYP with a primary diagnosis of SEN may also present with coexisting mental health conditions, such as low mood or anxiety, in order to provide these children with the support they need to prevent further deterioration of their mental health, and subsequent attendance to ED in suicidal crisis. Not only will this ease distress and potentially save lives, but will also reduce the substantial burden placed on healthcare services [55].

In the group of children who presented to the ED in a suicidal crisis with self-harm, significant associations were found between sex, self-harm, and method of self-harm. Specifically, females were more likely to have self-harmed, and the most common method of self-harm involved self-poisoning (i.e., overdose). No sex differences were found among other means of self-harm (e.g., cutting, suffocating, etc.). Such evidence of higher rates of self-harm in females aligns with the previous literature [56,57,58], and highlights the need for greater support for this group. Furthermore, a high percentage of CYP who previously received input from CAMHS were re-admitted to the ED for a suicidal crisis, thus indicating that those children were another particularly vulnerable group in need of further support.

The present study has the merit of contributing to the existing literature on suicidal crises in CYP. However, it also presents some limitations that need to be addressed by future research; therefore the results provided should be interpreted with caution. First, this study is based on the data from only one hospital in the North-West of England, which limits the generalisability of the results obtained. Multicentre studies involving hospitals from different part of the UK are warranted to strengthen the results of the present research. Second, the sample size was relatively modest, which may have limited the statistical power. Third, the sample of children included in this study is limited to those who attended hospital, meaning they either were severely in crisis or decided to reach out for help. Thus, the results may not be representative of the whole population of CYP experiencing suicidal crisis. The hospital’s policies also mean that only CYP up to the age of 16 were included in this study, and so ACEs that are typically more prevalent in 16–18-year-olds may have been missed. Furthermore, given the nature of this study, no control group was included; this would have allowed for comparative measures of those variables that seem to be implicated in increasing the risk of suicidal crisis. Finally, this was a retrospective study, and as such, some information is missing. Part of the data was also collected during the COVID-19 pandemic (discussed in further detail in [4]), so may not be representative of typical ED attendance. Despite the above limitations, this study provided valuable insight into the key factors associated with suicidal crises in young people, findings which may be beneficial for health and education providers to help decrease the proportion of CYP experiencing crisis.

## 5. Conclusions

To summarise, the presence of ACEs is associated with ED attendance for the suicidal crisis in CYP. Additionally, female sex, history of self-harm, mental health difficulties, and previous input from CAMHS were also associated with repeat hospital attendances. Our findings have implications for early identification of and intervention with children who may be at a heightened risk for ACEs and associated suicidal crises, in order to both prevent young people from experiencing difficulties and to ease pressures on health services. However, further work is still needed on a larger scale with longitudinal datasets to confirm our findings.

## Figures and Tables

**Table 1:** Demographic details of patients attending Emergency Department (ED) in suicidal crisis.

Demographics	Attendance at ED for Suicidal Crisis during Data Collection Period (n; % of Total Sample)	Previously Attended ED for Suicidal Crisis before Current Data Collection Period (n; % of Total Sample)
***All***	240; 100%	58; 24%
***Sex***		
Female	160; 66.6%	45; 18.8%
Male	80; 33.3%	12; 5.0%
***Age (mean; range)***	13.5 (9–15)	13.7 (8–16)
***Ethnicity***		
White British	222; 92.5%	51; 21.3
Other	16; 6.7%	5; 2
Unknown	2; 0.8%	1; 0.5
***Previous mental health issues***		
Anxiety	43; 17.9%	10; 4.2%
Anxiety comorbidities	10; 4.2%	3; 1.3%
Anxiety low mood	9; 3.8%	4; 1.7%
Low mood	40; 16.7%	20; 8.3%
Low mood comorbidity	5; 2.1%	3; 1.3%
other	35; 14.6%	10; 4.2%
No mental ill health	98; 40.4%	7; 2.9%
***SEN***		
Attention Deficit Hyperactivity Disorder (ADHD)	12; 5.0%	7; 2.9%
ADHD other learning disabilities	3; 1.3%	0; 0%
Autism spectrum condition (ASC)	21; 8.8%	8; 3.3%
ASC and ADHD	12; 5.0%	5; 2.1%
ASC, ADHD and learning disabilities	1; 0.4%	0; 0%
ASC and learning disabilities	3; 1.3%	0; 0%
Learning disabilities	6; 2.5%	1; 0.4%
No SEN	182; 75.8%	36; 15.0%
***ASC traits***		
yes	51; 21.3%	16; 6.7%
no	189; 78.8%	41; 17.1%
***Previously known to CAMHS***		
yes	154; 64.2%	55; 22.9%
no	86; 35.8%	2; 0.8%
***Currently under CAMHS***		
yes	54; 22.5%	25; 10.5%
no	186; 77.5%	32; 13.3%
***History of self-harm***		
yes	162; 67.5%	55; 22.9%
no	78; 31.6%	2; 0.8%

Variable Descriptors: Previous_A & E—Attended the ED for suicidal crisis prior to the data collection period; History_DSH—Known to have previously deliberately self-harmed; Known_CAMHS—Previously known to Child and Adolescent Mental Health Services; Low_Mood—Previously received a diagnosis of low mood/depression; Age—Age at time of ED attendance; ASD_Traits—Suspected to have autistic traits; Diagnosis_Other—Previously received a mental health diagnosis not listed; Open_CAMHS—Currently open to Child and Adolescent Mental Health Services; Pathway—The clinical pathway the patient was referred on to; ASD—Previously received an autism diagnosis; ADHD—Previously received an ADHD diagnosis; Anxiety—Previously received an anxiety diagnosis; Social_Worker—Currently known to a social worker; Neglect—Known to be experiencing neglect in the home; Siblings—Known to have siblings; Abuse—Known to be experiencing abuse in the home; Domestic_Violence—Known to be living in a home with domestic violence; Other_Home_Situation—Living in a home situation not listed; Single_Parent—Living with a single parent; Step_Parent—Living with a parent and a stepparent; Sep_Loss_Parent—Separated from or lost a parent; Means—If self-harmed, the means with which they self-harmed; Risk—Clinician determined risk to life score (Pierce score); Gender—CYP reported gender; Comorbidities—Diagnosed with multiple mental health conditions; Suspect_SH—Suspected to have self-harmed; Social_Problem—Attendance coded as “social problem” on hospital system; Parental_Mental_health—Parent reported own mental health issues; Parent_Drug_Misuse—Parent reported own drug misuse; Parental_Crime—Parent reported own engagement in crime.

## Data Availability

The data analysed in this study are sensitive and confidential. Requests to access these datasets should be directed to EA.
